# The Orphan Nuclear Receptor Gene NR0B2 Is a Favorite Prognosis Factor Modulated by Multiple Cellular Signal Pathways in Human Liver Cancers

**DOI:** 10.3389/fonc.2021.691199

**Published:** 2021-05-14

**Authors:** Runzhi Zhu, Yanjie Tu, Jingxia Chang, Haixia Xu, Jean C. Li, Wang Liu, Ahn-Dao Do, Yuxia Zhang, Jinhu Wang, Benyi Li

**Affiliations:** ^1^ The Children’s Hospital, Zhejiang University School of Medicine, National Clinical Research Center for Child Health, Hangzhou, China; ^2^ Zhejiang University Cancer Center, Hangzhou, China; ^3^ Department of Urology, The University of Kansas Medical Center, Kansas City, KS, United States; ^4^ Department of Pharmacology, Toxicology & Therapeutics, The University of Kansas Medical Center, Kansas City, KS, United States

**Keywords:** NR0B2, liver cancer, survival, PI3K - AKT pathway, MAPK (ERK1/ERK2)

## Abstract

**Background:**

Liver cancer is a leading cause of cancer death worldwide, and novel prognostic factor is needed for early detection and therapeutic responsiveness monitoring. The orphan nuclear receptor NR0B2 was reported to suppress liver cancer development in a mouse model, and its expression levels were reduced in liver cancer tissues and cell lines due to hypermethylation within its promoter region. However, it is not clear if NR0B2 expression is associated with cancer survival or disease progression and how NR0B2 gene expression is regulated at the molecular level.

**Methods:**

Multiple cancer databases were utilized to explore NR0B2 gene expression profiles crossing a variety of human cancers, including liver cancers, on several publicly assessable bioinformatics platforms. NR0B2 gene expression with or without kinase inhibitor treatment was analyzed using the qPCR technique, and NR0B2 protein expression was assessed in western blot assays. Two human hepatocellular carcinoma cell lines HepG2 and Huh7, were used in these experiments. NR0B2 gene activation was evaluated using NR0B2 promoter-driven luciferase reporter assays.

**Results:**

NR0B2 gene is predominantly expressed in liver tissue crossing human major organs or tissues, but it is significantly downregulated in liver cancers. NR0B2 expression is mostly downregulated in most common cancers but also upregulated in a few intestinal cancers. NR0B2 gene expression significantly correlated with patient overall survival status in multiple human malignancies, including lung, kidney, breast, urinary bladder, thyroid, colon, and head-neck cancers, as well as liposarcoma and B-cell lymphoma. In liver cancer patients, higher NR0B2 expression is associated with favorite relapse-free and progression-free survival, especially in Asian male patients with viral infection history. In addition, NR0B2 expression negatively correlated with immune infiltration and PIK3CA and PIK3CG gene expression in liver cancer tissues. In HepG2 and Huh7 cells, NR0B2 expression at the transcription level was drastically reduced after MAPK inhibition but was significantly enhanced after PI3K inhibition.

**Conclusion:**

NR0B2 gene expression is altered mainly in most human malignancies and significantly reduced in liver cancers. NR0B2 is a prognosis factor for patient survival in liver cancers. MAPK and PI3K oppositely modulate NR0B2 expression, and NR0B2 gene upregulation might serve as a therapeutic responsiveness factor in anti-PI3K therapy for liver cancer.

## Introduction

Liver cancer is the fifth (male) or seventh (female) most common cause of cancer death now in the United States (1). However, liver cancer has a much higher incidence in Africa and Asia, and its fatality rate has been on the rise in the past two decades worldwide (2). The incidence of liver cancer is about 2-7 times more in men compared to women depending on their geographical area, but the mechanism behind this sexual dimorphism is still unclear (3). Although male hormone and its cognate androgen receptor (AR) were considered as significant contributors (3), clinical trials with antiandrogen or anti-AR treatment did not yield a favorite outcome (4).

Hepatocellular carcinoma (HCC) is the predominant form of liver cancer, and chronic viral infection from hepatitis B/C viruses (HBV/HCV) has been the major risk factor (5). On the other hand, chronic liver inflammation due to metabolic syndrome and nonalcoholic fatty liver disease after long-term high-calorie food intake is becoming the prime causes for HCC incidence in western countries (6). However, it is conceivable that genetic/ethnic diversity also contributes to the difference in HCC pathogenesis. Understanding the molecular risk factors in HCC development and progression will be critical for improving early detection and developing effective targeted therapies to combat this deadly disease.

NR0B2 (nuclear receptor subfamily 0 group B member 2), also called small heterodimer partner (SHP), is an orphan nuclear receptor without a conventional zinc-finger DNA binding domain (7). It acts as a transcriptional repressor by binding to other nuclear receptors to regulate various metabolic pathways, including glucose, bile acid, cholesterol, and fatty acid homeostasis in the organs of the liver, pancreas, and kidney (8). It was reported that NR0B2 gene expression was significantly reduced in liver and kidney cancers and that overexpression of NR0B2 protein suppresses liver cancer development, indicating it is a tumor suppressor (9, 10). However, it is not clear if NR0B2 expression is also altered in other human cancer types and if the alteration is associated with patient disease history or outcome. In addition, there are only very few reports related to the involvement of cellular signaling pathways in the regulation of NR0B2 gene expression in cancer cells.

In this study, we sought to investigate if NR0B2 gene expression is altered crossing the spectrum of human cancers. We utilized multiple publicly available bioinformatic platforms to obtain and to analyze the data of clinical parameters, gene expression profiles, and pathological diagnosis. Our study revealed that NR0B2 gene expression is mainly downregulated in many common cancers, while its upregulation is only seen in fewer cancer types. Survival data showed that NR0B2 expression is a favorite prognosis factor in patients with liver, kidney, lung, urinary bladder cancers but is a negative factor in patients with colon, thyroid, uterine, and head-neck cancers. Patient stratify analysis revealed that NR0B2 expression is a favorite factor in liver cancer patients of Asian males with viral infection history. Correlation analysis discovered that NR0B2 expression is negatively correlated with PI3K pathway genes PIK3CA and PIK3CG in liver cancer tissues. Consistently, PI3K inhibition significantly enhanced NR0B2 expression at the transcription level in human liver cancer HepG2 and Huh7 cells. These data demonstrated NR0B2 as a prognosis factor in human cancer with a diverse clinical significance. It is feasible that NR0B2 expression might serve as a biomarker for anti-PI3K therapeutic responsiveness in human liver cancers.

## Materials and Methods

### Cell Lines, Culture Condition, and Experimental Reagents

Human HCC HepG2 cell line was obtained from ATCC (Manassas, VA), and Huh7 cell line was obtained from Health Science Research Resources Bank (JCRB0403, Osaka, Japan). Cells were cultured in DMEM media with 10% fetal bovine serum (FBS) and 1% penicillin and streptomycin at 37°C in a 5% CO_2_ setting. All kinase-selective small chemical inhibitors for MAPK, MEK1/2, PI3K JNK, and p38MAPK, as well as GW4064, were purchased from Cayman Chemicals (Ann Arbor, MI). Chemicals were initially dissolved in DMSO and then diluted with cell culture media into the final concentration at a 1000-fold dilution. Chemical treatment time and final concentrations was indicated in the figure legends.

Antibodies for NR0B2 (clone N2C3) were obtained from GeneTex (Irvine, CA). Antibodies for ERK (clone 137F5), phosphor-ERK (clone D13.14.4E), AKT (clone 40D4), phospho-AKT (clone D9E), FXR (clone E4B8P), and Actin (clone E4D9Z) were purchased from Cell Signaling Tech (Danvers, MA). HRP-conjugated secondary antibodies and luminol reagents were purchased from Santa Cruz Biotech (Dallas, TX).

### Western Blot, qPCR, and Luciferase Reporter Assays

For western blot assays, cells were harvested in cold PBS solution, and protein lysates were extracted using RIPA buffer as described (11, 12). After protein assay, equal amounts of proteins from each treatment were subjected to western blot assay with the antibodies indicated in the figure.

For real-time quantitative RT-PCR (qPCR) assays, cells were harvested in cold PBS solution, and total RNAs were extracted using the TRIzol™ reagent (Invitrogen). After the cDNA synthesis, the qPCR reaction was conducted with an SYBR Green-based PCR master mix (Bio-Rad) described in our publications (13, 14). The house-keeping gene GAPDH was used as an internal control for data normalization.

For luciferase assay, cells were seeded in a 6-well plate overnight and then transfected with an empty reporter construct pGL3 or the human NR0B2 promoter-driven luciferase construct (hSHP-LUC) as described in our publication (14). After treatment with kinase inhibitors or GW4064 overnight, cells were harvested for luciferase measurement, and the final readings were normalized with the relative levels of total proteins, as described in our publication (15).

### Bioinformatic Data Platforms

Oncomine™ data processing platform (www.oncomine.org) was used to obtain gene expression data derived from cDNA microarray analysis, and the comparison between cancer and its normal counterpart tissues was conducted to generate a fold-change ratio.

Tumor Immune Estimation Resource (TIMER™, cistrome.shinyapps.io/timer) is a multi-modular platform for comprehensive analysis (16, 17). Its Gene module provides visualization of target gene expression and tumor infiltration level of immune cells. Its Diff Exp module also provides gene expression comparison between tumor and adjacent normal tissues for any gene of interest across all TCGA tumors. Its Correlation module conducts a Spearman analysis of a pair of the interested genes in each cancer type.

The Kaplan-Meier Plotter (kmplot.com) is a meta-analysis tool to assess the effect of gene expression at the mRNA, miRNA, and protein levels on patient survival outcomes in 21 types of human cancers (18). The database was built on the GEO, EGA, and TCGA resources. All available datasets for human cancers were processed for NR0B2 gene expression. The survival data with statistical significance were downloaded for presentation and discussion.

The PrognoScan (PrognoScan.org) is a database built for analyzing the prognostic significance of a candidate gene in an extensive collection of publicly available cancer microarray datasets with clinical outcomes such as overall survival and disease-free survival (19). It uses the minimum *P*-value approach to define an optimal cut-point in gene expression levels for survival comparison.

### Data Presentation and Statistical Analysis

All experiments were carried out in triplicates and repeated two or three times. The real-time RT-PCR results and luciferase data are presented as the mean plus the stand error of the mean (SEM) from three separate experiments. The luciferase assay results are shown as fold induction compared to the DMSO control. The images from the western blot assay were representative of multiple blots. Statistical analysis for the differences between groups was carried out using the Student *t*-test (SPSS software, Chicago, IL), and the p < 0.05 was considered significant.

## Results

### NR0B2 Gene Is Aberrantly Expressed in Multiple Human Cancers

**Figure 1:**
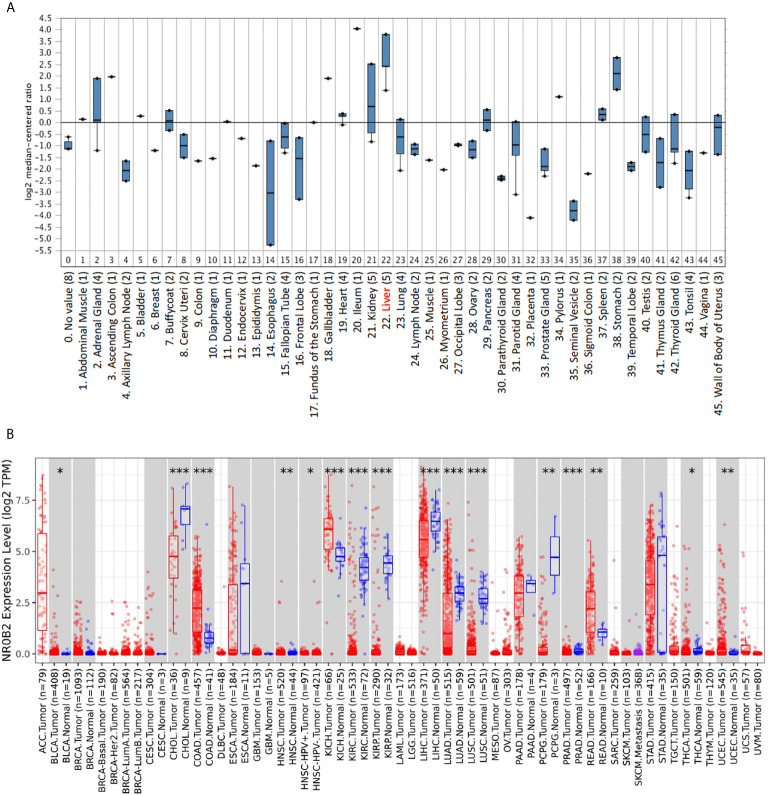
NR0B2 expression profiles in benign and malignant tissues. **(A)** NR0B2 gene expression profiles in human tissues were queried from the Shyamsundar cDNA microarray dataset (20) in the Oncomine database. Box plot was generated based on the log_2_ median-centered ratio in each organ/tissue type. **(B)** The differential profiles of NR0B2 gene expression between malignant and adjacent benign tissues were queried from the TCGA tumor database on the Tumor Immune Estimation Resource (TIMER) platform (16, 17). Data are displayed using box plots. The statistical significance of differential expression was analyzed using the Wilcoxon test. *p < 0.05, **p < 0.01, ***p < 0.001.

To obtain a complete image of the NR0B2 gene expression pattern in human organs/tissue, we re-analyzed the microarray data collected by the Oncomine database. Two large datasets were available for NR0B2 gene expression containing 45 (20) (**Figure 1A**) and 95 (11) (**Figure S1**) benign tissues, respectively. Both datasets showed that liver tissues expressed the highest levels of NR0B2 gene, followed by kidney, adrenal gland, stomach, spleen, and heart. These data are in agreement with the published literature in the field that the primary function of NR0B2 gene-coded protein SHP is in bile acid metabolism, innate immune response, and gene regulation (7, 21–23).

We then compared NR0B2 gene expression in cancer tissues with their matched benign counterparts. The Oncomine database has more than 30 datasets showing a significant reduction of NR0B2 gene expression in human cancers, including liver, renal, lung, and gastric carcinomas (**Table S1**). Meanwhile, about ten datasets showed a significant increase of NR0B2 gene expression in human cancers, such as the esophagus and colorectal adenocarcinoma, ovarian serous surface papillary carcinoma, and brain medulloblastoma (**Table S2**).

To verify the NR0B2 gene expression pattern obtained from the Oncomine database, we used a secondary dataset from the TCGA collection. We found a similar expression pattern (**Figure 1B**), NR0B2 gene downregulation in liver, kidney, and lung carcinomas but upregulation in the colon and rectal adenocarcinomas. These data suggest that NR0B2 gene expression is differently regulated in various human organs/tissues and that the aberrant NR0B2 expression in human cancers is opposite to the patterns in their benign counterpart tissues.

### NR0B2 Expression Is a Favorite Prognosis Factor in Liver Cancers

**Figure 2:**
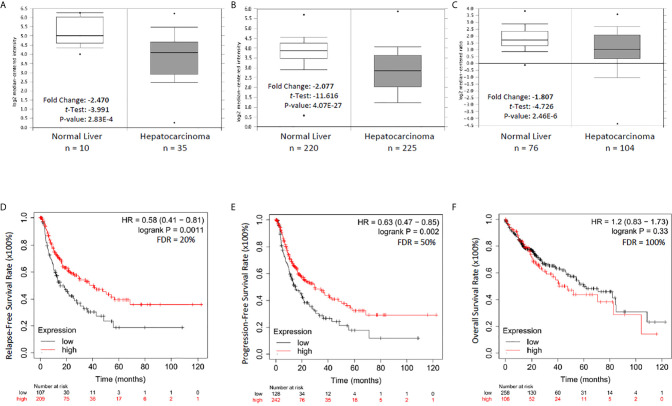
NR0B2 expression is significantly reduced and associated with recurrent-free and progression-free survival in liver cancer patients. **(A–C)** NR0B2 expression data were queried from three representative datasets in the Oncomine™ database; A-Wurmbach (24), B-Roessler (25), and C-Chen (26). Statistical information is inserted in the plot, and the case numbers for each group were listed below the plot. **(D–F)**. The Kaplan Meier plotter was used to assess the effect of NR0B2 gene expression (RNAseq data for the mRNA level) on liver cancer patients’ survival status (n = 364). The data sources for the analysis include GEO, EGA, and TCGA. The prognostic value of NR0B2 gene expression levels was compared by splitting the patient cohort into two groups according to the quantile expressions of the NR0B2 gene. The Kaplan-Meier survival plot was used to calculate the hazard ratio with 95% confidence intervals and log-rank *p*-value.

To explore the clinical significance of NR0B2 gene expression in human cancers, we analyzed the correlation between patient survival and NR0B2 gene expression, with an emphasis on liver cancers. As shown in **Figures 2A–C**, NR0B2 gene expression is reduced about 1.8-2.5 folds in hepatocarcinoma tissues compared to the benign counterparts. Survival analysis revealed that higher NR0B2 expression is significantly associated with a favorite recurrent-free (**Figure 2D**) and progression-free (**Figure 2E**) survival in a cohort of 316-370 patients. Although NR0B2 expression had no significant correlation with overall survival (**Table 1**), the higher NR0B2 expression level was significantly associated with worse overall survival in early-stage (HR = 2.84), well-differentiated cancers (HR = 2.94), as well as alcohol consumption (HR = 2.18) and male patients without viral hepatitis history (HR = 1.9). Interestingly, higher NR0B2 expression is a favorite factor for overall survival in patients with viral hepatitis history (**Figure 2F**), especially for those without alcohol consumption and Asian male patients. These data were supported by a previous report that SHP protein suppressed HCV replication in human liver cancer HuH7 cells (27).

**Table 1:** The Kaplan Meier plotter was used to calculate the hazard ratio with 95% confidence intervals and log-rank *p*-value after stratifying the cohort into different subgroups based on the clinical and pathological parameters.

high vs low comparison	cases	HR (95%CI)	p value	FDR
overal surival	364	1.2 (0.83-1.73)	n.s.	100%
disease-specific survival	357	1.3 (0.82-2.08)	n.s.	100%
stage-1	170	1.24 (0.67-2.29)	n.s.	100%
stage-2	83	2.84 (1.23-6.54)	**0.0103**	50%
stage-3	83	0.62 (0.32-1.21)	n.s.	100%
stage-4	3	n.a.	n.a.	n.a.
grade-1	55	2.94 (1.09-7.94)	**0.026**	50%
grade-2	174	1.63 (0.97-2.74)	n.s.	100%
grade-3	118	0.49 (0.23-1.05)	n.s.	100%
grade-4	4	n.a.	n.a.	n.a.
AJCC-T2	90	2.2 (1.05-4.63)	**0.033**	>50%
AJCC-T3	78	0.76 (0.4-1.42)	n.s.	100%
AJCC-T4	13	n.a.	n.a.	n.a.
Vascular invasion –none	203	0.71 (0.42-1.2)	n.s.	100%
Vascular invasion –micro	90	1.8 (0.84-3.38)	n.s.	100%
Vascular invasion -macro	14	n.a.	n.a.	n.a.
Male	246	0.73 (0.47-1.15)	n.s.	100%
Female	116	1.67 (0.91-3.06)	n.s.	100%
White	184	1.56 (0.97-2.51)	n.s.	100%
Asian	155	0.54 (0.29-1.02)	n.s.	100%
Black	17	n.a.	n.a.	n.a.
Alcohol consumption-yes	117	2.18 (1.14-4.14)	**0.0151**	>50%
Alcohol consumption-no	202	0.75 (0.42-1.32)	n.s.	100%
Hepatitis virus-yes	150	0.52 (0.27-0.99)	**0.0416**	>50%
Hepatitis virus-no	169	1.38 (0.87-2.2)	n.s.	100%
Alcohol consumption-no				
plus Hepatitis virus-yes	111	0.3 (0.12-0.8)	**0.0103**	50%
Male Hepatitis virus-yes	153	0.44 (0.21-0.89)	**0.0199**	>50%
Male Hepatitis virus-no	96	1.9 (1.01-3.58)	**0.044**	>50%
Asian Hepatitis virus-yes	93	0.33 (0.13-0.85)	**0.0157**	>50%
Asian Hepatitis virus-no	49	1.56 (0.64-3.84)	n.s.	100%

Highly significant parameters were highlighted in bold font. n.s., not signiificant.

**Figure 3:**
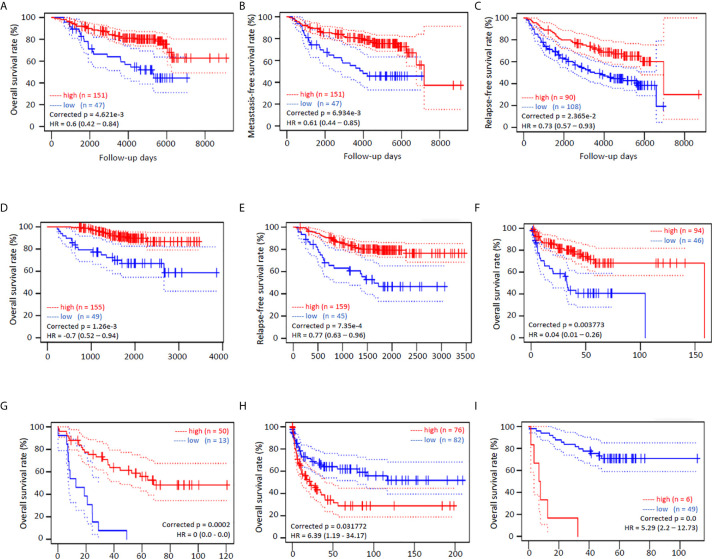
NR0B2 expression is significantly associated with patient survival in multiple human cancers. Kaplan-Meier plots were generated on the PrognoScan platform (19). NR0B2 expression by cDNA microarray analysis in cancer tissues was dichotomized into two groups, high (red) or low (blue), at the optimal cut-point by the minimum *p*-value approach (19). Survival curves were plotted as solid lines with 95% confidence intervals for each group by dotted lines. Correct p and HR values (95% CI) are inserted in the plots. **(A–C)** NR0B2 expression with HG-U133A microarray chips in 198 breast cancer cases (GSE7390) (28). **(D, E)** NR0B2 expression with HG-U133plus2 microarray chip in 204 stage I-II lung adenocarcinoma cases (GSE21210) (29). **(F)** NR0B2 expression with HG-U133A microarray chips in 140 liposarcoma cases (GSE30929) (30). **(G)** NR0B2 expression with HG-U133plus2 microarray chip in 63 uveal melanoma cases (GSE22138) (31). **(H)** NR0B2 expression with HG-U133A microarray chips in 158 B-cell lymphoma cases (GSE4475) (32). **(I)** NR0B2 expression with HG-U133plus2 microarray chip in 55 colon cancer cases (GSE17537) (33).

We also analyzed the survival significance of NR0B2 expression in breast, lung, colon, eye, and soft tissue cancers. Higher NR0B2 expression is significantly associated with a favorite overall survival, metastasis-free and relapse-free survival in breast cancer patients (**Figures 3A–C**). Higher NR0B2 expression levels were significantly associated with favorite overall and relapse-free survival (**Figures 3D, E**). Interestingly, higher NR0B2 expression levels were also a favorite prognostic factor in liposarcoma (**Figure 3F**) and eye uveal melanoma patients (**Figure 3G**). In agreement with a significant upregulation of NR0B2 gene expression in colon and B-cell lymphoma (**Table S2**), higher NR0B2 expression levels were significantly associated with worse overall survival in these patients (**Figures 3H, I**). These correlations of NR0B2 expression with patient overall survival status for renal cancers and lung cancers were consistent with the data obtained from a different bioinformatic platform except in breast cancer patients, as shown in **Figure S2**. In addition, NR0B2 is a favorite survival factor in bladder cancers but a worse factor in thyroid cancers, lung squamous cancers, uterine corpus endometrial cancers, and head-neck squamous cancers. These data suggest that NR0B2 plays a diverged role in different human cancers.

### NR0B2 Expression Is Negatively Associated With Tumor-Infiltrating Lymphocytes and PI3K Genes in Liver Cancers

**Figure 4:**
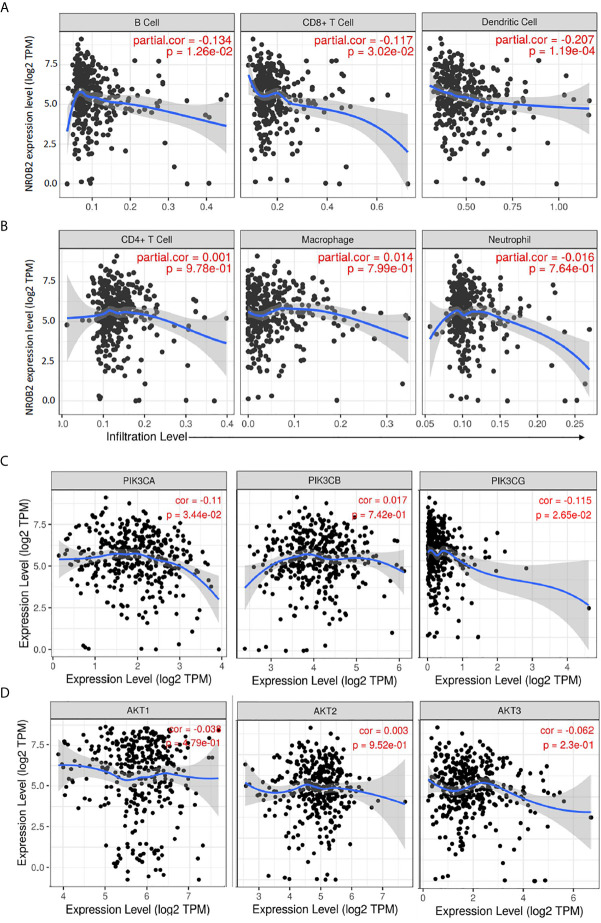
Correlation analysis between NR0B2 expression and tumor-infiltrating immune cells, as well as PI3K/AKT gene expression in liver cancers. **(A, B)** The Gene module on the Tumor Immune Estimation Resource (TIMER) platform was used to visualize the correlation between NR0B2 gene expression and immune infiltration levels in liver cancer tissues (16, 17). Scatterplots show a negative correlation of NR0B2 expression with tumor-infiltrating B-cell, CD8^+^ T cell, and dendritic cell but not CD4^+^ T cell, macrophage, and neutrophil. The partial Spearman’s rho value and statistical significance were inserted in the plot. **(C, D)** The Correlation module on the Tumor Immune Estimation Resource (TIMER) platform was used to visualize the correlation between NR0B2 gene expression and class IA PI3K genes in liver cancer tissues (16, 17). Scatterplots show a negative correlation of NR0B2 expression with PIK3CA and PIK3CG gene expression but not with PIK3CB and ATK1-3 genes. The Spearman’s rho value and statistical significance were inserted in the plot.

Recently, we and others reported that NR0B2 expression suppressed inflammation (13, 34, 35) and innate immune response (36). We, therefore, analyzed the correlation between NR0B2 expression and tumor-infiltrating lymphocytes using the TIMER database. Our analysis discovered a partial but significant correlation between lower NR0B2 expression levels and higher tumor infiltration of B cells, CD8^+^ T cells, and dendritic cells (**Figure 4A**). NR0B2 expression has no correlation with other tumor-infiltrating lymphocytes, including CD4^+^ T cells, macrophages, and neutrophils (**Figure 4B**). These data are consistent with recent reports that NR0B2 is a negative regulator of host immune response.

PI3K/AKT pathway was recently identified as cancer biomarkers in liver cancer patients, especially for those patients with viral infection history (37). Our data mining analysis revealed a significant but negative correlation of gene expression between the NR0B2 gene and two class IA PI3K genes PIK3CA and PIK3CG (**Figure 4C**). NR0B2 gene expression had no significant correlation with PIK3CB and AKT genes (**Figure 4D**). These data are in agreement with a recent report showing a negative correlation between FXR/NR0B2 action and PI3K pathway in liver regeneration (38).

### NR0B2 Expression Is Differently Modulated by MAPK and PI3K Pathways in Liver Cancer Cells

**Figure 5:**
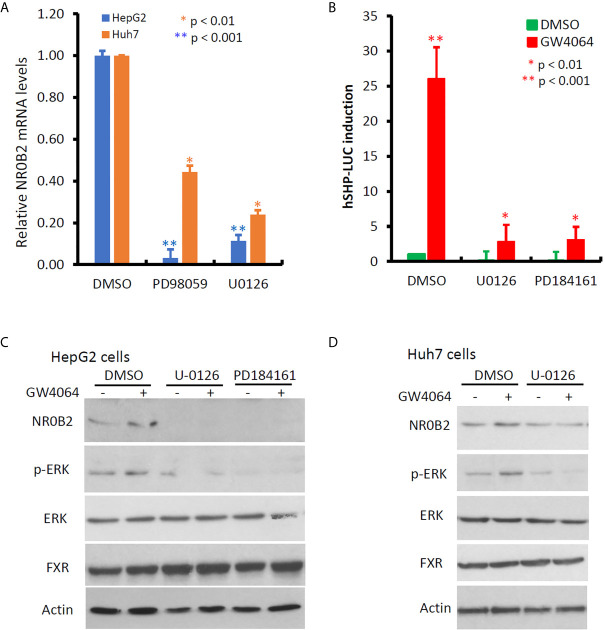
MAPK activity is essential for NR0B2 gene expression in HCC cells. **(A)** HepG2 and Huh7 cells were treated with DMSO or MAPK inhibitors PD98059 (50 μM) and U0126 (10 μM) for 24 h. Total RNAs were extracted for qPCR assays. The relative expression levels of NR0B2 gene expression are calculated against the DMSO treatment (set as 1.0). Error bar indicates the SEM from three independent experiments. The asterisk indicates a significant difference compared to the DMSO control (Student *t*-test). **(B)** HepG2 cells were seeded in 6-well plates and then transfected with human NR0B2 promoter-driven luciferase reporter construct (hSHP-LUC, 0.5 μg DNA/well). Cells were pre-treated with MEK inhibitors U0126 (10 μM) and PD184161 (10 μM) for 30 min and then stimulated with GW4064 (5 μM) for 24 h. Luciferase assay was carried out as described in our publication (14, 15). The relative reporter activity was calculated against the DMSO control (set as 1). Error bar indicates the SEM from three independent experiments. The asterisk indicates a significant difference compared to the DMSO control (Student *t*-test). **(C)** HepG2 cells were pre-treated with U0126 (10 μM) and PD184161 (10 μM) for 30 min, followed by GW4064 (5 μM) stimulation for 24 h. Cells were harvested for western blot assays with the antibodies as indicated. Actin blot served as the protein loading control. **(D)** Huh7 cells were pre-treated with U0126 (10 μM) for 30 min, followed by GW4064 (5 μM) stimulation for 24 h. Cells were harvested for western blot assays with the antibodies as indicated. Actin blot served as the protein loading control.

NR0B2 expression is mainly regulated by NR1H4 gene-encoded FXR protein and other co-factors (23, 39, 40). Meanwhile, cellular signal pathways including MAPK, JNK, and PI3K were involved in regulating NR0B2 expression after growth factor, metformin, or bile acid stimulation (41, 42). To explore the cellular pathways involved in regulating NR0B2 gene expression, we first used a pharmacological approach coupled with a qPCR technique for endogenous NR0B2 mRNA levels. Kinase-specific inhibitors for MAPK kinase (PD98059 and U0126), JNK kinase (SP600125), p38 kinase (SB203580), and PI3K (LY294002) were used to treat liver cancer cells HepG2 and Huh7, followed by qPCR-based NR0B2 mRNA measurement. As shown in **Figure 5A**, MAPK inhibitors drastically suppressed NR0B2 expression in both cell lines, with a predominantly strong effect on HepG2 cells. Next, we verified this effect with an NR0B2 promoter-driven luciferase reporter assay. MAPK kinase inhibitors (U0126 and PD184161) blocked the basal reporter activity and abolished bile acid analog GW4064-induced reporter activity (**Figure 5B**). Thirdly, we confirmed the MAPK involvement in modulating NR0B2 protein levels by western blot (**Figures 5C, D**). Consistently, GW4064 stimulated MAPK pathway activation as evidenced by ERK phosphorylation (**Figures 5C, D**). MAPK inhibition did not affect FXR protein levels. These data demonstrated the essential requirement of MAPK activity in NR0B2 expression at the transcriptional level.

**Figure 6:**
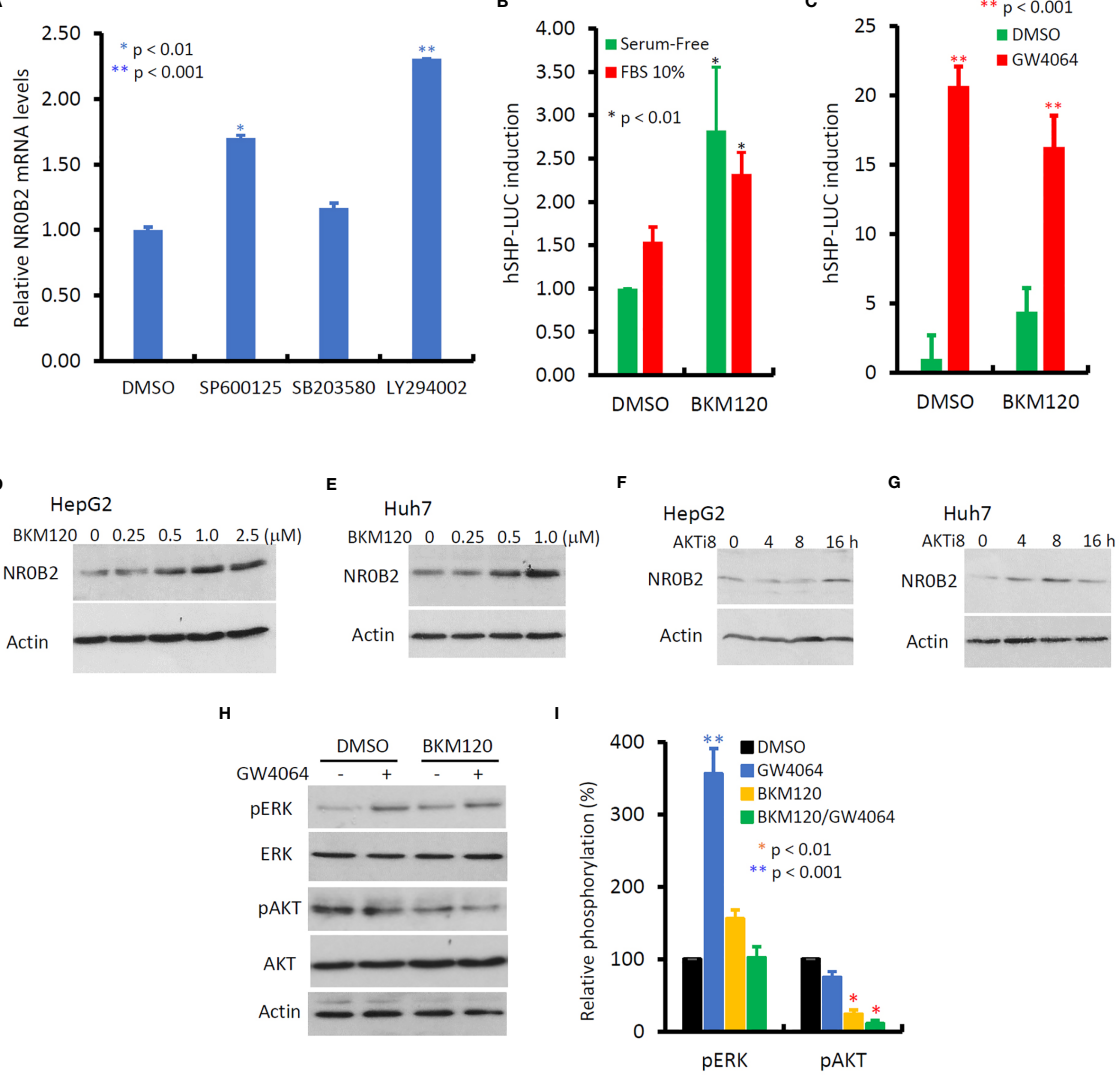
Inhibition of PI3K activity enhances NR0B2 gene expression in HCC cells. **(A)** HepG2 cells were treated with DMSO or JNK inhibitor SP600125 (50 μM), p38MAPK inhibitor SB203582 (10 μM), and PI3K inhibitor LY294002 (50 μM) for 24 h. Total RNAs were extracted for qPCR assays. The relative expression levels of NR0B2 gene expression are calculated against the DMSO treatment (set as 1.0). Error bar indicates the SEM from three independent experiments. The asterisk indicates a significant difference compared to the DMSO control (Student *t*-test). **(B)** HepG2 cells were seeded in 6-well plates and then transfected with human NR0B2 promoter-driven luciferase reporter construct (hSHP-LUC, 0.5 μg DNA/well). After serum starvation overnight, cells were pre-treated with PI3K inhibitor BKM120 (10 μM) for 30 min and then cultured with serum-free or 10% FBS for 24 h. Luciferase assay was carried out as described in our publication (14, 15). The relative reporter activity was calculated against the DMSO control (set as 1). Error bar indicates the SEM from three independent experiments. The asterisk indicates a significant difference compared to the DMSO control (Student *t*-test). **(C)** HepG2 cells were seeded in 6-well plates and then transfected with human NR0B2 promoter-driven luciferase reporter construct (hSHP-LUC, 0.5 μg DNA/well). After pretreatment with BKM120 (10 μM) for 30 min, cells were stimulated with GW4064 (5 μM) for 24 h. Luciferase assay was carried out as described earlier. **(D–G)** HepG2 or Huh7 cells were treated with DMSO, BKM120 for 24 h at the indicated concentrations or AKT inhibitor 8 (AKTi8, 10 μM) for the indicated period. Cells were harvested for western blot assays with the antibodies as indicated. **(H, I)** HepG2 cells were pre-treated with BKM120 (10 μM) for 30 min, followed by GW4064 (5 μM) stimulation for 24 h. Cells were harvested for western blot assays with the antibodies as indicated. Band density for phosphorylated ERK or AKT were normalized against total ERK or AKT, respectively. Each treatment’s relative density was calculated against the DMSO control (set as 100%) individually and then plotted as a bar graph. Data represent three independent experiments. The asterisk indicates a significant difference compared to the DMSO control (Student *t*-test).

In contrast to MAPK inhibitors, PI3K and JNK inhibitors vastly enhanced NR0B2 expression (**Figure 6A**), of which PI3K inhibitor had a more profound effect in HepG2 cells. This PI3K inhibition-induced NR0B2 upregulation was further explored in NR0B2 promotor-driven luciferase reporter assays. A novel PI3K inhibitor BKM120 significantly enhanced basal and serum-stimulated but not GW4064-induced NR0B2 reporter activity (**Figures 6B, C**). This enhancing effect was also evidenced at the protein levels after BKM120 or AKT inhibitor treatment at both dose- and time-dependent manner (**Figures 6D G**). Although BKM120 treatment slightly increased ERK phosphorylation levels, it did not affect GW4064-stimulated ERK phosphorylation (**Figures 6H, I**). Conversely, GW4064 treatment moderately reduced AKT phosphorylation, which was further reduced by BKM120 treatment, as expected. These data indicate that bile acid-induced NR0B2 expression requires ERK but not PI3K/AKT activity and that PI3K/AKT activity prevents NR0B2 expression at the basal condition.

## Discussion

In this study, we re-analyzed several public datasets for NR0B2 expression patterns in human benign and malignant tissues and investigated the involvement of MAPK and PI3K pathways in human liver cancer cells. Our data revealed that NR0B2 gene expression was highly expressed in liver, kidney, and gastric tissues but was significantly reduced in malignant tissue derived from these organs. Meanwhile, lung adenocarcinomas exerted a downregulation, but colorectal adenocarcinomas upregulated NR0B2 expression. Survival analysis showed that higher NR0B2 expression levels were associated with better survival status in liver, lung, breast, soft tissue (liposarcoma), and eye cancers. Conversely, higher NR0B2 expression is a worse prognosis factor in colon cancers and B-cell lymphomas. NR0B2 expression is conversely correlated with tumor-infiltrating B-cells, CD8^+^ T cells, and dendritic cells, as well as PIK3CA and PIK3CG gene expression in liver cancer tissues. Gene expression analysis determined that the ERK pathway was essential for basal and GW4064-induced NR0B2 expression while the PI3K/AKT pathway only prevented NR0B2 expression at the basal but not bile acid stimulation condition in liver cancer cells.

There is a paucity of NR0B2 expression from human cancer specimens, and so far, only two reports showed NR0B2 downregulation in 10 cases of liver cancers (HCC) (14) and 24 cases of renal cancers (RCC) (10). With the advance of bioinformatic technologies, many public databases are assessable to analyze gene expression profiles in a variety of human cancers. This study took advantage of these bioinformatic resources and systemically explored the NR0B2 expression profiles in benign and malignant tissues. Our data confirmed the predominant expression profile of the NR0B2 gene in benign liver and kidney tissues. Interestingly, NR0B2 expression was significantly downregulated in malignant tissues derived from these organs, consistent with previous reports (10, 14, 43), indicating a dramatic alteration in regulating NR0B2 gene expression after malignant transformation. Higher NR0B2 expression is associated with fewer disease relapse and progression in liver cancer patients and is also associated with a favorite overall survival prognosis in human breast cancers, lung adenocarcinomas, liposarcomas, and eye uveal melanomas. However, higher NR0B2 expression is a worse survival factor in colon cancers and B-cell lymphomas.

Currently, NR0B2 gene regulation is not fully clear, especially in human cancers. It was reported that the NR0B2 gene promoter region is hypermethylated in human liver cancer tissues and cell lines, and treatment of liver cancer cells with DNA demethylation agent 5-Aza-2′-deoxycytidine vastly enhanced NR0B2 expression (14). Besides, the PI3K/AKT pathway was upregulated in liver cancers (37), and PI3K/AKT inhibitors have been utilized as an anticancer agent for liver cancer treatment (44). Interestingly, in this study, we found that NR0B2 expression negatively correlated with the expression levels of two PI3K genes PIK3CA and PIK3CG, while PI3K inhibition significantly enhanced NR0B2 expression in liver cancer cells. These data suggest that PI3K/AKT pathway overactivation during liver cancer development or progression might be a potential mechanism for NR0B2 downregulation.

In this study, we discovered the opposite effect of ERK inhibition on NR0B2 gene expression than PI3K inhibition. It was reported that ERK kinase activity is essential for the basal but not FGF15-stimulated NR0B2 gene expression in mouse liver (41). We found that ERK activity is essential for NR0B2 gene expression at the basal and GW4064-stimulated conditions. As a FXR agonist, GW4064 was reported to have a mixed effect on ERK phosphorylation/activation. It was shown to enhance ERK phosphorylation in bone marrow-derived macrophages (42) but to suppress ERK phosphorylation in rat vascular smooth muscle cells (45), human colon cancer SNU-C4 cells (46), and liver cancer HLE cells (47). In this study, GW4064 treatment increased ERK phosphorylation in HepG2 and Huh7 cells, in parallel with a complete blockage of NR0B2 expression at the transcriptional level. These data suggest that GW4064 caused a cell-specific effect on ERK phosphorylation/activation. Considering a previous report showing ERK activity as a critical factor for NR0B2 protein stability in HepG2 cells (48), we hypothesize that in human liver cancer cells, ERK activity might be essential for both NR0B2 gene expression and protein stability.

Tumor-infiltrating immune cells are the major parts of the tumor microenvironment associated with disease progress, immunotherapy response, and patient survival (49, 50). We and others reported that NR0B2 has a unique function in suppressing inflammation and innate immunity in response to liver cell injury (13, 21, 35, 36). This study discovered a negative correlation of NR0B2 expression with tumor-infiltrating lymphocytes, including B cells, CD8^+^ T cells, and dendritic cells in liver cancer tissues. Because inflammatory response and tumor-infiltrating lymphocytes have diverse functions in tumor progression and anti-tumor immunity (21, 50), the clinical significance of this negative correlation between NR0B2 expression and tumor-infiltrating lymphocytes needs more investigation.

Chronic viral hepatitis is a substantial contributing factor for liver cancer development and progression (51). HCV infection was shown to increase NR0B2 gene expression in human liver cells (52), and NR0B2 gene silencing or an excessive overexpression all reduced HCV replication in Huh7 cells (27). This study found an interesting correlation of NR0B2 expression with over survival in liver cancer patients with viral hepatitis history (HR = 0.52, p = 0.0416), which was more significant in Asian male patients (HR = 0.33, p = 0.0157). These data indicate that higher NR0B2 expression has a protective effect in viral hepatitis-related liver cancers. Further investigation with a large population is needed to verify this correlation.

In conclusion, in this study, we discovered that NR0B2 expression is predominantly downregulated in multiple malignant tissues and upregulated in few cancers. NR0B2 expression is a favorite factor in human cancers from the liver, kidney, lung, urinary bladder, breast, eye, and soft fat tissues, but is a worse factor in colon, thyroid, uterine, and head-neck cancers, as well as B-cell lymphoma. Especially, NR0B2 expression is a favorite survival factor in Asian male patients with viral infection-related liver cancers. NR0B2 is also negatively correlated with PIK3CA and PIK3CG genes in liver cancer tissues, and PI3K inhibition enhances NR0B2 gene expression in liver cancer cells. Further investigation is needed to verify the clinical significance of NR0B2 expression in protecting viral infection-related liver cancer development and progression.

## Data Availability Statement

The original contributions presented in the study are included in the article/**Supplementary Material**. Further inquiries can be directed to the corresponding authors.

## Author Contributions

RZ and BL designed the study. RZ, JL, and JW performed the analysis of bioinformatics data from public databases. JC, YT, HX, JL, WL, A-DD, and YZ performed experiments. BL and YZ performed the statistical analysis and generated figures and tables. RZ and BL drafted the manuscript. All authors contributed to the article and approved the submitted version.

## Funding

JL was a recipient of the travel award from Geographical Management of Cancer Health Disparities Program (GMP) Region 3 Travel Funds in 2018. NIH R01DK119131 to YZ.

## Conflict of Interest

The authors declare that the research was conducted in the absence of any commercial or financial relationships that could be construed as a potential conflict of interest.

## Supplementary Material

Supplementary Figure 1 NR0B2 gene expression profiles in human tissues were queried from the Roth cDNA microarray dataset (11) in the Oncomine database. Box plot was generated based on the log_2_ median-centered ratio in each organ/tissue type.

Supplementary Figure 2 The overall survival profiles were analyzed with the Kaplan-Meier Plotter based on NR0B2 expression in multiple human cancers. The hazard ratio with 95% confidence intervals and log-rank p-value and case numbers in each group were inserted in the plot.

Supplementary Table 1 NR0B2 gene expression down-regulation in human cancers. Comparative data of NR0B2 gene expression profiles in human malignant and benign counterpart tissues were obtained from the Oncomine database.

Supplementary Table 2 NR0B2 gene expression up-regulation in human cancers. Comparative data of NR0B2 gene expression profiles in human malignant and benign counterpart tissues were obtained from the Oncomine database.
