# Supplementary figures and images for: The Orphan Nuclear Receptor Gene NR0B2 Is a Favorite Prognosis Factor Modulated by Multiple Cellular Signal Pathways in Human Liver Cancers
Source: Front Oncol. 2021 May 14;11:691199. doi: 10.3389/fonc.2021.691199 (PMC8162207; doi:10.3389/fonc.2021.691199)

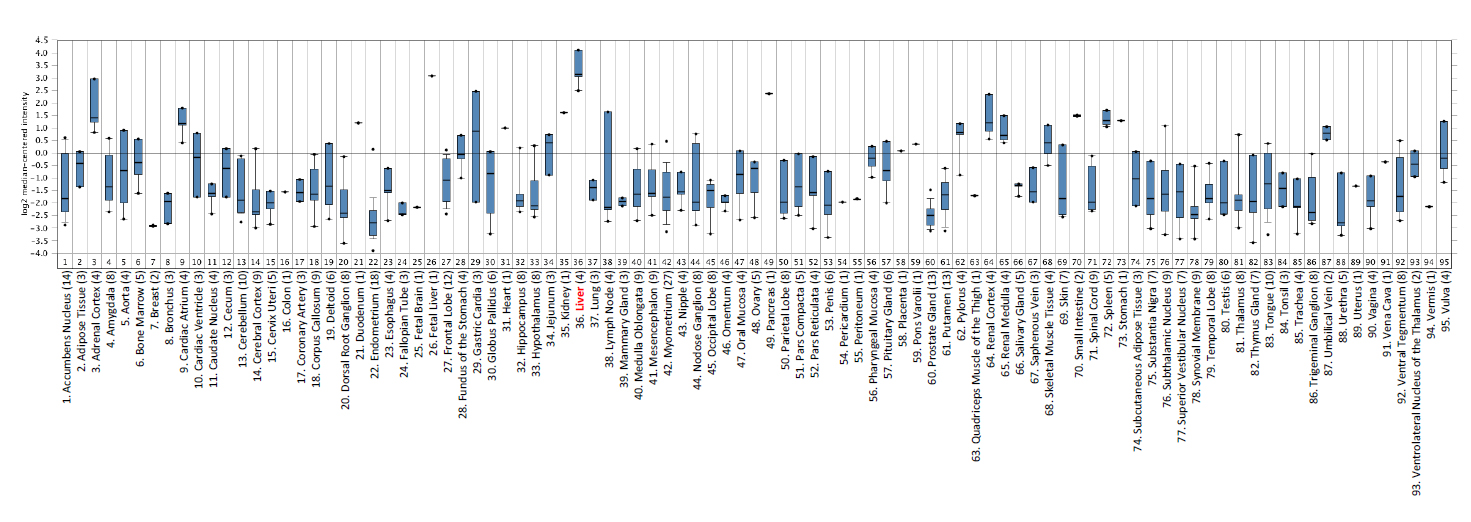

Supplement: Supplementary Figure 1 — NR0B2 gene expression profiles in human tissues were queried from the Roth cDNA microarray dataset (11) in the Oncomine database. Box plot was generated based on the log2 median-centered ratio in each organ/tissue type. [file Image_1.jpeg]

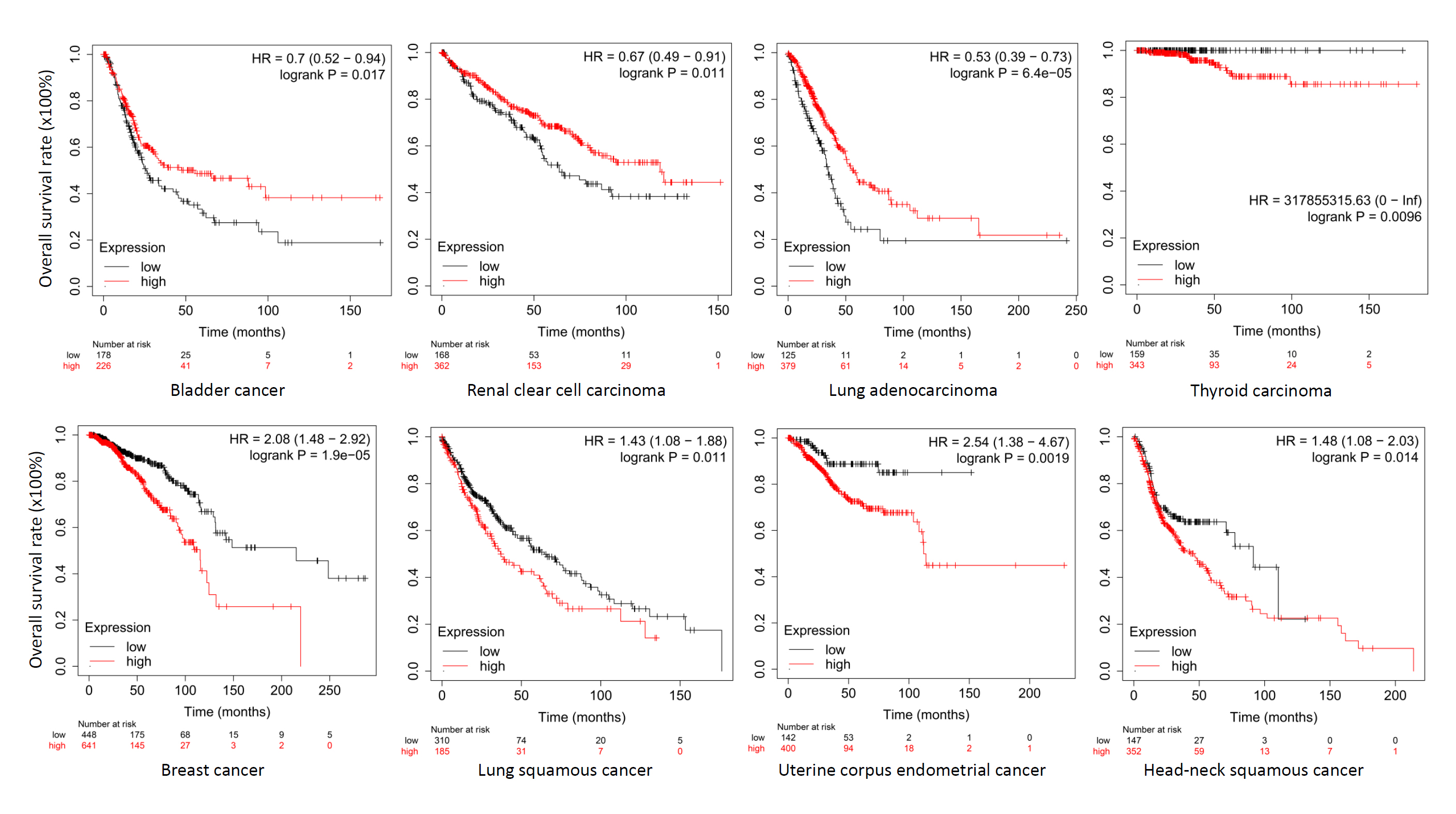

Supplement: Supplementary Figure 2 — The overall survival profiles were analyzed with the Kaplan-Meier Plotter based on NR0B2 expression in multiple human cancers. The hazard ratio with 95% confidence intervals and log-rank p-value and case numbers in each group were inserted in the plot. [file Image_2.jpeg]

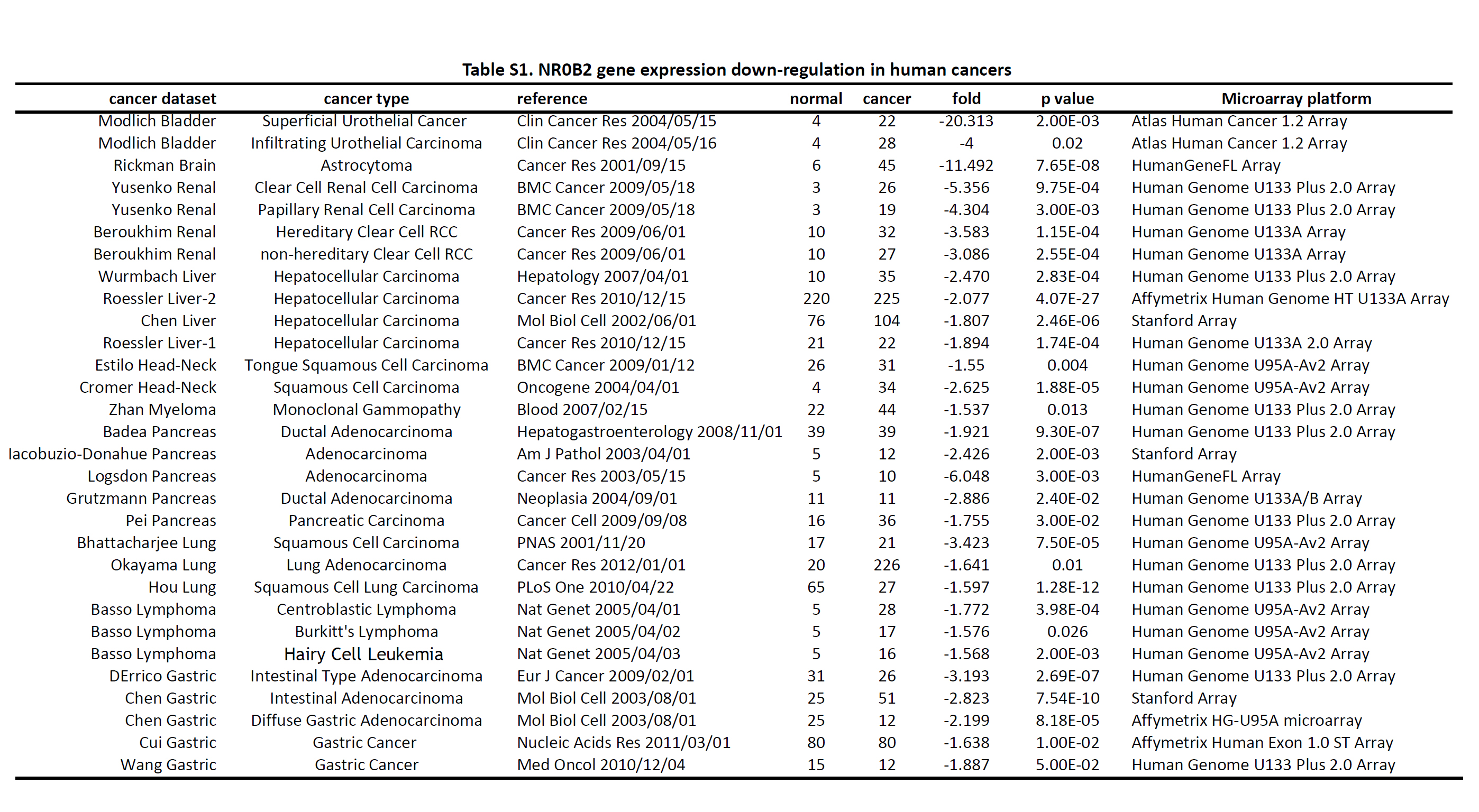

Supplement: Supplementary Table 1 — NR0B2 gene expression down-regulation in human cancers. Comparative data of NR0B2 gene expression profiles in human malignant and benign counterpart tissues were obtained from the Oncomine database. [file Image_3.jpeg]

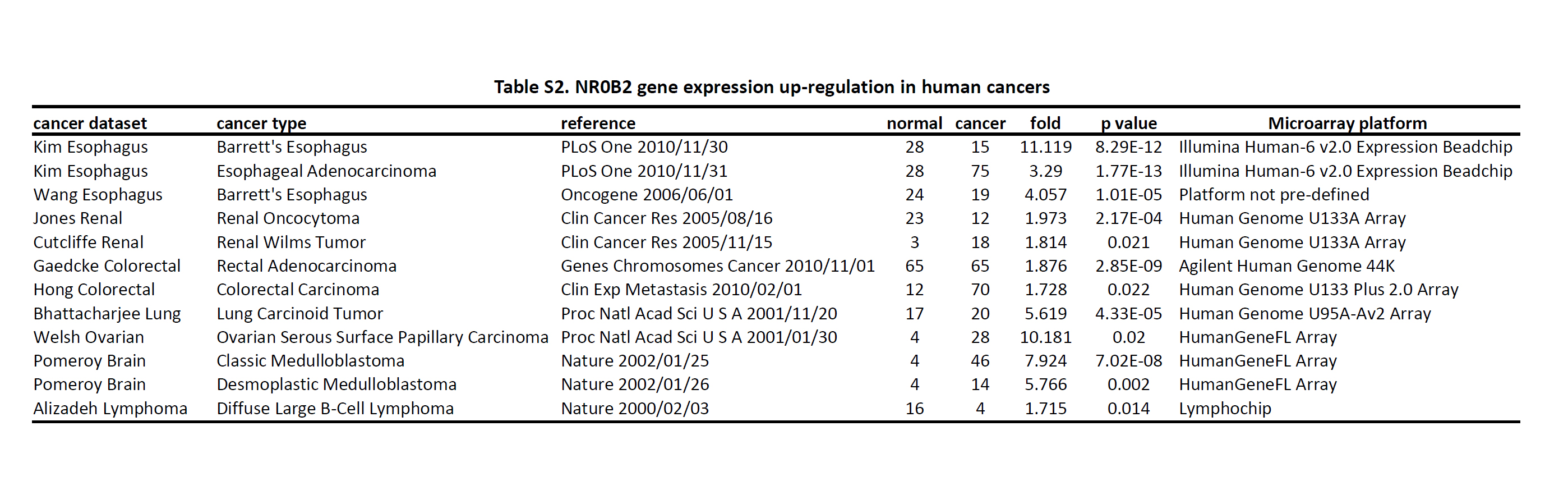

Supplement: Supplementary Table 2 — NR0B2 gene expression up-regulation in human cancers. Comparative data of NR0B2 gene expression profiles in human malignant and benign counterpart tissues were obtained from the Oncomine database. [file Image_4.jpeg]
